# Antidiabetic, Anticholinesterase and Antioxidant Activity vs. Terpenoids and Phenolic Compounds in Selected New Cultivars and Hybrids of Artichoke *Cynara scolymus* L.

**DOI:** 10.3390/molecules24071222

**Published:** 2019-03-28

**Authors:** Igor Piotr Turkiewicz, Aneta Wojdyło, Karolina Tkacz, Paulina Nowicka, Francisca Hernández

**Affiliations:** 1 Department of Fruit, Vegetable and Plant Nutraceutical Technology, Wrocław University of Environmental and Life Sciences, 37 Chełmońskiego Street, 51-630 Wrocław, Poland; igor.turkiewicz@upwr.edu.pl (I.P.T.); karolina.tkacz@upwr.edu.pl (K.T.); paulina.nowicka@upwr.edu.pl (P.N.); 2 Department of Plant Sciences and Microbiology, Plant Production and Technology Group, Escuela Politécnica Superior de Orihuela, Miguel Hernández University de Elche, Ctra.de Beniel, km 3.2, 03312 Orihuela, Alicante, Spain; francisca.hernandez@umh.es

**Keywords:** artichoke, sugar, organic acid, polyphenolics, carotenoids, chlorophylls, identification, LC/MS-QTof

## Abstract

The aim of the study was to analyze the chemical composition of new artichoke cultivars and hybrids and to assess their potential health-promoting properties. Polyphenols, carotenoids and chlorophylls were identified by liquid chromatography-photodiode detector-mass spectrometry/quadrupole time of flight (LC-PDA-MS/QTof) and quantified by ultra performance liquid chromatography-photodiode detector (UPLC-PDA). Sugar and organic acid profiles were prepared, and antioxidant capacity (ABTS, FRAP and ORAC) and in vitro antidiabetic (inhibition of α-amylase and α-glucosidase) and anticholinesterase (inhibition of acetylcholinesterase (AChE) and butyrylcholinesterase (BuChE)) potentials were evaluated. The analyses revealed a highly varied content of bioactive compounds and great health-promoting potential significantly depending on a cultivar. The content of polyphenolic compounds ranged from 1681.49 (“Sambo”) to 3638.91 (“Symphony”) mg/100 g of dry weight (DW). “Blanca de Tudela” contained the highest amounts of carotenoids and chlorophylls (3761.91 mg/100 g DW) and exhibited high ABTS and ORAC capacity. Inhibition of antidiabetic enzymes was significant in cvs. “Symphony” and “Calico”. Additionally, all tested cultivars and hybrids turned out to be effective inhibitors of neurodegenerative enzymes.

## 1. Introduction

Artichoke (*Cynara scolymus* L.) belongs to the Asteraceae family and is one of the oldest plants cultivated by humans. It has been used as a dietary and medicinal product since 4th century B.C. This perennial crop, typically cultivated in the Mediterranean basin, is widespread throughout the world [1]. The global area of artichoke cultivation in 2016 was 124,900 ha, of which more than 50% was in Europe. Leading producers of artichoke in 2016 included Italy (365,991 tons), Egypt (236,314 tons), and Spain (185,796 tons) [2]. Edible parts of artichoke comprise an immature inflorescence (called capitula or head), receptacle, and bracts [3,4]. Artichoke represents an essential ingredient of the Mediterranean diet and can be eaten as a fresh, boiled, steamed, fried or canned vegetable [1,3]. The bracts are a rich source of inulin, fibers and minerals but also of bioactive compounds, such as polyphenols [5].

The previously mentioned edible parts of artichoke constitute only 15–20% of total biomass of the plant. Remaining wastes are unsuitable for consumption, however, they are a rich source of inulin (which is a prebiotic) and polyphenolic compounds [6,7]. Artichoke residues from food industry can also be used as biofuels, thus treating them as a solid waste is uneconomical [7]. To solve the problem of competition between the food industry and the pharmaceutical and/or cosmetic industry, it seems appropriate to use parts of artichoke that are unsuitable for the market for the recovery of bioactive compounds for non-food applications.

Several pharmacological experiments have demonstrated the health promoting effects of artichoke extracts including: hepatoprotective, cholagogic and choleretic [1]; antidyspeptic and antispasmodic [8]; anti-inflammatory, hypoglycemic, antiatherogenic and antihypercholesterolemic [9]; and antioxidant and anti-tumor [9]. In addition, artichoke extracts have shown antimicrobial and probiotic [4] activity.

Polyphenol content and composition strongly depend on a plant part, genotype, growing stage and processing conditions [5,10]. For example, their accumulation in the bracts increases from the outside towards the inner parts of the plant. The levels of biologically active compounds are also influenced by plant cultivar and maturity [11].

Numerous clinical trials confirmed that consumption of fruit and vegetables, typically of the Mediterranean diet, reduces the risk of chronic non-communicable diseases such as cancer and cardiovascular disorders [12]. Compared with other vegetables, artichoke contains high levels of polyphenols, which are responsible for health promoting effects [1].

Nutritional characteristics of artichoke perfectly match the current interest in a healthy lifestyle and balanced diet. People are looking for a way to supplement their daily diet with nutrients, minerals and vitamins. They are, however, no longer interested in tablets or powders, but seek traditional food products that enrich their meals with essential ingredients positively affecting bodily functions. Artichokes can fulfill these expectations. In this paper we show that due to its composition and properties artichoke could become one of the essential components of a balanced diet and serve as functional food.

The primary aim of this study was to compare: (i) basic chemical composition (dry weight (DW), soluble solid content (SSC), sugar and organic acid content); (ii) the content of bioactive compounds such as polyphenolics, carotenoids and chlorophylls (identification and quantification analyzed by LC-PDA-MS/QTof and UPLC-PDA, respectively); and (iii) biological properties (antioxidant, antidiabetic, and anticholinesterase activity) in the inner bracts of selected new cultivars and hybrids of artichoke. Our secondary aim was to determine the relationships between the basic chemical composition, polyphenolic and terpenoid compounds and specific biological activities of selected new cultivars and hybrids of artichoke. Research literature contains no reports on the effects of artichoke extracts on anticholinesterase activity. In addition, research on antidiabetic properties of specific cultivars and hybrids described in this paper have not been reported by the authors.

## 2. Results

### 2.1. Basic Chemical Composition of Artichoke

Dry matter content (Table 1) of the artichoke inner bracts differed significantly (*p* < 0.05) from 13.74% (“Sambo”) to 15.79% (“Blanca de Tudela”). Rouphael et al. [13] reported dry weight content of 19 artichoke cultivars most commonly grown in Europe as ranging from 9.1% to 10.6%, with 10.2% for cv. “Blanca de Tudela”. DM content in artichoke cultivars and hybrids cultivated in Italy fell between 10.7% and 16.9% (average 13.2%) [14]. Differences in values obtained by different authors may be due to many factors, e.g., cultivar, climatic conditions, agrotechnical techniques or maturity stage [15].

Soluble solids content (SSC) in juice obtained from artichoke bracts reached 10.30 and 11.50°Bx (*p* < 0.05) in cvs. “Blanca de Tudela” and “Calico”, respectively. Thus far, there have been no reports on SSC in artichoke but the values are similar in orange (11.30°Bx), apple (10.80°Bx) or black currant (12.60°Bx) juice [16]. “Symphony” and “Opera” were the objects with the highest sugar content of 0.69 and 0.67 g/100 g FW, respectively (*p* < 0.05), while “Blanca de Tudela”, “Calico” and “Sambo” ranked at the other end of the scale (0.31; 0.32 and 0.38 g/100 g FW, respectively). The main identified saccharide was glucose, followed by sucrose and fructose. Glucose was also the most abundant sugar in the analyzed new cultivars and hybrids except for cv. “Calico”, where sucrose dominated. In other hybrids with the highest sugar content (“Symphony” and “Opera”), glucose represented 67.5% and 63.4% of total carbohydrates, respectively. Nicoletto et al. [17] reported total sugar content in artichoke to range between 0.22 and 0.36 g/100 g FW. Contrary to that, Petropoulos, Pereira, Ntatsi, Danalatos, Barros and Ferreira [10] identified sucrose as a dominant sugar in Greek artichoke genotypes, followed by glucose and fructose. Total sugar content in artichoke is similar to that of spinach (0.53 g/100 g FW), but lower than in broccoli (0.70 g/100 g FW), lettuce (1.62 g/100 g FW), or green pepper (1.93 g/100 g FW) [18].

The content of organic acids (*p* < 0.05) equaled 1.53 and 3.05 g/100 g FW in “Symphony” and “Calico”, respectively. Adipic acid was the most abundant organic acid in all investigated objects, followed by malic (except for “Symphony”), quinic (except for “Blanca de Tudela”, “Calico” and “Sambo”), and citric acid. The remaining acids, i.e., oxalic, maleic, shikimic, succinic and formic, were at a similarly low level of 0.01 to 0.09 g/100 g FW. The research literature does not contain many reports on determination of organic acid content in artichoke. Nicoletto, Santagata, Tosini and Sambo [17] investigated the levels of malic and oxalic acid in Italian cultivars, where they ranged from 0.10 to 0.11 g/100 g FW and 0.02 to 0.04 g/100 g FW, respectively. Apart from different aromatic compounds, sugars and organic acids are crucial for organoleptic characteristics of fruit and vegetables. Organic acids affect also their quality and nutritional values [19].

### 2.2. Identifications and Quantification of Terpenoids as Carotenoids and Chlorophylls by LC-PDA-MS/QTof

**Figure 1:**
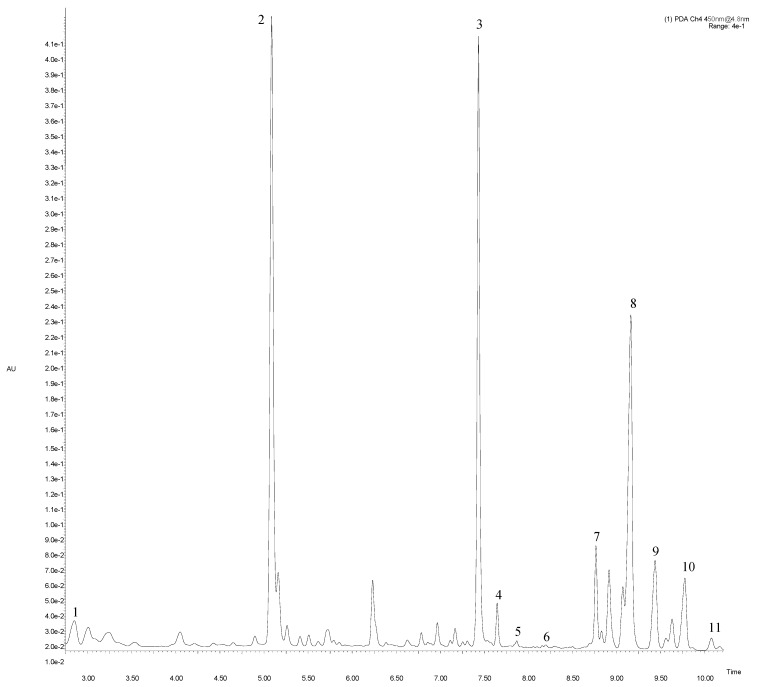
Main fragment of UPLC-PDA chromatogram at 450 nm of artichoke “Calico” cv. Peak number identities are display in Table 2.

Carotenoid compounds were identified by comparison with commercial standards, by their detectable UV–Vis spectra and by their MS spectra obtained in a positive ion mode only. The analysis revealed 11 compounds belonging to carotenoids and chlorophylls, with high content of β-carotene, chlorophyll a and pheophytin a. Carotenoids (Table 2, Figure 1) were identified based on the fragment ions at *m*/*z* 601.54 for neoxanthin and *m*/*z* 537.54 for β-carotene, as described by Wojdyło et al. [20]. A compound with [M + H]^+^ at *m*/*z* 551.15 co-eluted with the standard was identified as lutein. Two of three identified carotenoids (neoxantin and lutein) were eluted before β-carotene.

A peak with [M + H]^+^ at *m*/*z* 907.67 and fragmentation ions at *m*/*z* 780.63/629.10 deprived of phytyl chain (C_20_H_38_) was identified as chlorophyll b. Chlorophyll a with [M + H]^+^ at 892.69 and MS/MS fragments (615.13/555.34) was consistent with results obtained by Kolniak-Ostek [21]. According to Delpino-Rius et al. [22], compounds with Rt at 7.66 and 9.50 min were identified as pheophytin b and b’. Those two derivatives showed identical [M + H]^+^ at *m*/*z* 885.56 and a resulting product ion at *m*/*z* 553, but pheophytin b was eluted before pheophytin b’, similarly as described by Petrović et al. [23]. Based on literature data [24], a compound with molecular mass [M + H]^+^ at *m*/*z* 893.69 and fragmentation ions at *m*/*z* 615.13/555.34 corresponding to a loss of C_20_H_38_ (278 Da) and CH_3_COOC_20_H_39_ (338 Da), respectively, was identified as chlorophyll a’. The next peak was assigned to chlorophyll b’ with *m*/*z* at 907.69. 

Compounds with Rt at 9.81 and 10.09 min were identified as pheophytin a and a’. The corresponding epimers (pheophytin a and a’) of the main chlorophyll derivative (chlorophyll a) in the analyzed artichoke bracts showed identical [M + H]^+^ at *m*/*z* 871.72, but had different MS/MS fragmentation patterns (at *m*/*z* 593.20 and 623.36/593.20, respectively).

Carotenes are natural plant pigments found in almost all vegetable raw materials, and they serve as precursors of vitamin A. Chlorophylls belong to the least persistent plant colorants with a broad spectrum of pro-health properties. In addition to their important role in creating the color of fruit, they also have significant antioxidant capacity [25].

We found the highest concentration of carotenoids and chlorophylls in cv. “Blanca de Tudela” (510.13 and 3251.78 mg/kg DW), and the lowest in cv. “Calico” (208.68 and 948.72 mg/kg DW; *p* < 0.05). The most abundant carotene was β-carotene, followed by lutein and neoxanthin. Chlorophyll levels were cultivar independent and of the following order: chlorophyll a > pheophytin a’ > chlorophyll b > pheophytin a > pheophytin b > chlorophyll a’ (Table 2). Chlorophyll b’ and pheophytin b’ were the least abundant. For comparison, Guillén et al. [25] showed lower content of chlorophyll a and b and total content of chlorophylls in cv. “Blanca de Tudela” cultivated in Spain (355; 702, and 1058 mg/kg DW, respectively). Moreover, Romo-Hualde et al. [26] examined the content of lutein in artichoke cultivars grown in Navarra (Spain), and obtained lower values ranging from 0.53 to 1.47 mg/kg DW. This variability in the content of carotenoids and chlorophylls can be due to different methods of determination and differences between fresh and dry weight. According to Kolniak-Ostek [21], carotenoid and chlorophyll contents may depend on the cultivar and maturation stage.

### 2.3. Identification and Quantification of Polyphenols by LC-PDA-MS/QTof

**Figure 2:**
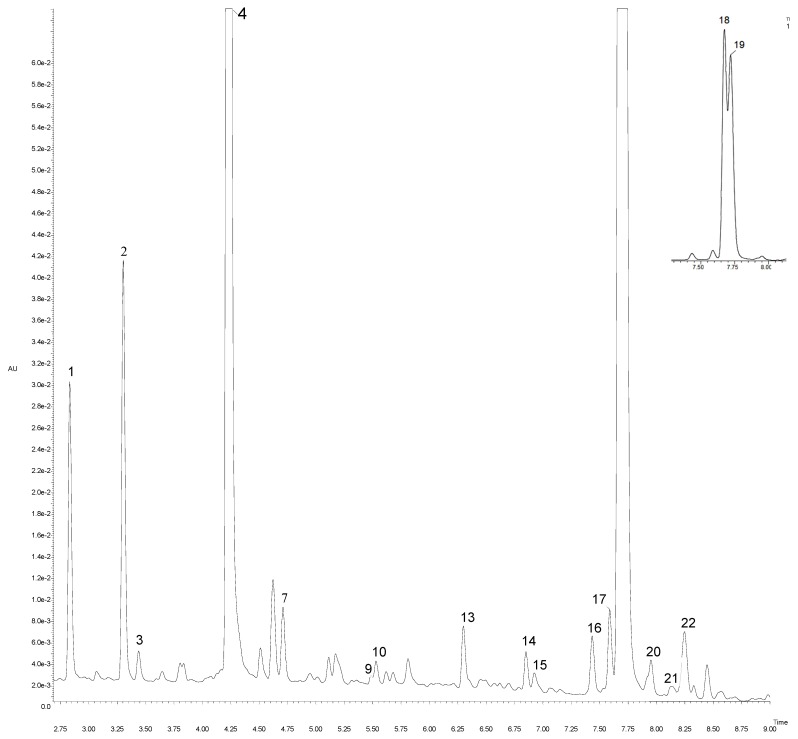
UPLC-PDA chromatogram at 320 nm of artichoke “Calico” cv. Peak number identities are displayed in Table 3.

We performed a qualitative analysis of the phenolic composition in the extracts from new artichoke cultivars and hybrids using LC-PDA-MS/QTof operating in negative ionization mode. In this work, we detected and characterized 25 phenolic compounds (Table 3, Figure 2). All the detected compounds were tentatively characterized by means of their detectable UV spectrum, MS data, together with observed MS/MS spectra in comparison with literature data.

In the group of hydroxycinnamic derivatives, we identified four monocaffeoylquinic isomers at different retention times (2.84, 3.31, 4.22 and 4.50 min). All compounds gave the same [M − H]^−^ at *m*/*z* 353.01 in accordance with the molecular formula C_16_H_17_O_9_. Their molecular ions showed a fragmentation pattern at *m*/*z* 190.99, which represents quinic acid and arises from a loss of C_9_H_7_O_3_ (163 Da). Therefore, according to Moglia et al. [27] and Sanchez-Rabaneda et al. [28], those compounds were identified as 1-*O*-caffeoylquinic acid (Rt = 2.84 min), neochlorogenic acid (Rt = 3.31 min), chlorogenic acid (Rt = 4.22 min), and cryptochlorogenic acid (Rt = 4.50 min). A compound with [M − H]^−^ at *m*/*z* 705.03 C_32_H_33_O_18_ and fragmentation ions at *m*/*z* 513.00/339.07/191.00 was tentatively identified as caffeoylquinic acid dimer. The other six compounds, with a precursor ion at *m*/*z* 515.01 and identical molecular formula C_25_H_23_O_12_, were identified as isomers of di-caffeoylquinic acid. Obtained MS/MS data demonstrated different fragmentation ions at *m*/*z* 353.00 (162 Da), 190.99 (162 Da) and 179.00 (12 Da), which indicated the fragmentation patterns typical for di-caffeoylquinic acids [28]. Compounds with Rt 4.90 and 6.30 min at *m*/*z* 367.00 have a characteristic MS/MS fragment ion at *m*/*z* 191.00, indicating the loss of quinic acid. Considering the results of Abu-Reidah et al. [29], we suggest that those two compounds are 3-*O*-feruloylquinic acid isomers. The next four compounds detected at Rt = 3.79, 5.48, 5.53 and 5.60 min, respectively, and [M − H]^−^ at *m*/*z* 513.05 (C_26_H_26_O_11_), gave an identical fragmentation pattern in the MS/MS spectra with a fragment ion at *m*/*z* 338.96 and at *m*/*z* 190.99. Therefore, in accordance with Deshpande et al. [30], they were proposed to be isomers of *p*-coumaroyl-feruloylquinic acid. Finally, a compound displaying a [M − H]^−^ ion at *m*/*z* 337.01 and MS/MS spectrum yielding a fragment ion at *m*/*z* 190.99 was tentatively identified as *p*-*coumaroylquinic acid*.

We characterized several compounds belonging to flavones. For example, a compound with Rt of 7.20 min, a precursor ion at *m*/*z* 461.09 and molecular formula C_21_H_19_O_11_ was, based on additional MS and MS/MS data, proposed as luteolin-7-glucuronide [10]. A compound with [M − H]^−^ at *m*/*z* 593.08 (C_27_H_29_O_15_) and a fragmentation ion at *m*/*z* 285.09, representing a natural loss of rutinoside moiety (308 Da), was proposed as luteolin-7-rutinoside (scolymoside), in accordance to Abu-Reidah, Arráez-Román, Segura-Carretero and Fernández-Gutiérrez [29] and Petropoulos et al. [31]. Compounds with Rt of 7.58 and 8.10 min were classified as apigenin-7-glucoside and apigenin-7-glucoronide. Those two derivatives showed [M − H]^−^ at *m*/*z* 431.09 and 445.09, respectively, and had an identical resulting product ion at *m*/*z* 269.00. Glucosides were eluted before glucuronides. The last two compounds detected at 8.44 and 8.49 min with [M − H]^−^ at *m*/*z* 596.01 and 543.02, respectively, displayed the same product ion at m/z 269.07 (ascribed to apigenin). This fragment ion comes from a subsequent splitting of acetyl groups (42 Da) and glucose moiety. Based on that and literature [29], those compounds were defined as isomers of apigenin. Total phenolic content, calculated as the sum of individual phenolic compounds, varied significantly between genotypes (*p* < 0.05), with “Symphony” displaying the highest (3638.91 mg/100 g DW), and “Sambo” the lowest content (1681.49 mg/100 g DW; Table 3). The main detected phenolic acids were caffeoylquinic acid derivatives, which was consistent with the findings of Negro et al. [32]. “Opera” and “Symphony” accumulated the greatest amounts of phenolic acids (3264.32 and 3437.64 mg/100 g DW), and in “Sambo” their content was the lowest (1567.60 mg/100 g DW). Flavonols were not particularly abundant in the new cultivars and hybrids in this study. The highest total concentration of flavonols was detected in “Symphony” (201.27 mg/100 g DW), and the lowest in “Sambo” (113.89 mg/100 g DW). According to Pandino et al. [33], the most abundant flavonol was luteolin-7-rutinoside (scolymoside), followed by apigenin-7-glucuronide. We found caffeoylquinic and di-caffeoylquinic acids to be the most abundant polyphenolic compounds in artichoke, which is consistent with other studies.

### 2.4. Analysis of Antioxidant, Antidiabetic and Anticholinesterase Activity

Total antioxidant activity in artichoke internal bracts ranged from 12.49 to 20.74 mmol Trolox and from 9.15 to 13.78 mmol Trolox/100 g for ABTS and FRAP assays, respectively (*p* < 0.05; Table 4). Cultivar “Blanca de Tudela” showed the highest and cv. “Calico” the lowest ABTS antioxidant activity. As per FRAP assay, it was, respectively, “Symphony” and “Blanca de Tudela”. Ferracane, Pellegrini, Visconti, Graziani, Chiavaro, Miglio and Fogliano [11] analyzed the effects of heat on ABTS antioxidant activity in artichoke and reported a lower value for raw inflorescence (2.07 mmol Trolox/100 g). In addition, Morales-Soto et al. [34], who investigated four cultivars of artichoke cultivated in Andalusia, documented lower values ranging from 0.67 to 1.45 mmol Trolox/100 g. Pandino, Lombardo, Mauromicale and Williamson [33] analyzed the polyphenolic profile and antioxidant properties using the FRAP method in artichoke cultivated in Sicily. For internal bracts their results varied from 0.75 to 2.84 mmol Trolox/100 g. Finally, Ferracane, Pellegrini, Visconti, Graziani, Chiavaro, Miglio and Fogliano [11] reported the values of antioxidant activity to reach from 5.69 to 6.75 mmol Trolox/100 g.

**Figure 3:**
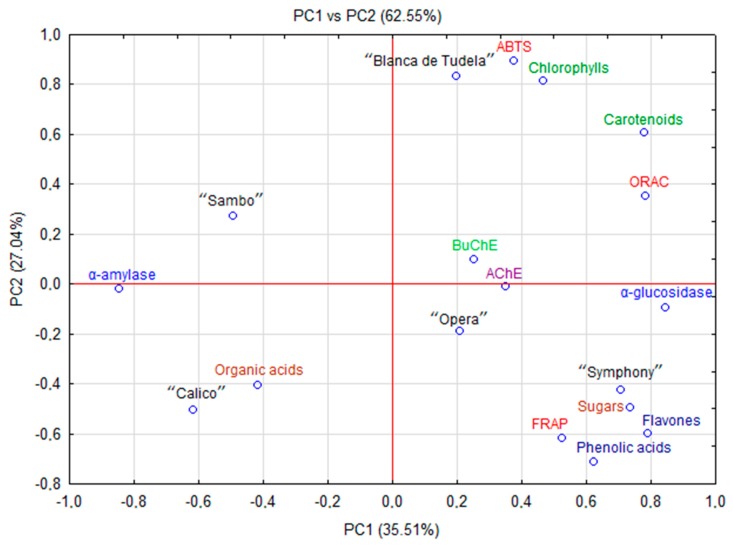
PCA scores plot showing the relationship among chemical parameters and biological activity of new varieties and hybrids of artichoke.

ORAC assay consists in measuring a decrease in fluorescence of a molecular probe due to chemical damage triggered by free radicals. This method is more sensitive than FRAP and used routinely in the food industry [35]. Our results for ORAC (*p* < 0.05) ranged from 16.80 (“Sambo”) to 39.98 mmol Trolox/100 g (“Blanca de Tudela”). Morales-Soto, García-Salas, Rodríguez-Pérez, Jiménez-Sánchez, de la Luz Cádiz-Gurrea, Segura-Carretero and Fernández-Gutiérrez [34] analyzed antioxidant properties of fruit and vegetables cultivated in Spain, and reported significantly lower values (0.45 to 0.99 mmol Trolox/100 g). They found that free radical scavenging ability of artichoke was lower than those of garlic, asparagus, zucchini or tomatoes (2.36, 2.40, 1.15 and 1.08 mmol Trolox/100 g, respectively), but higher than in potatoes or iceberg lettuce (0.26 and 0.58 mmol Trolox/100 g, respectively). Probably the differentiation between these plant materials results from the content of carotenes and chlorophylls because, based on the PCA, those compounds are mainly responsible for the ability of oxygen radical absorbance capacity in artichoke (Figure 3).

The antioxidant potential often depends on the content of bioactive compounds, especially phenolics (Pearson correlation between FRAP and phenolic acid = 0.671 and flavonols = 0.807, respectively). In artichoke this potential was reinforced by terpenoid compounds (Pearson correlation between ABTS or ORAC and carotenoids = 0.861 and 0.872; chlorophylls = 0.982 and 0.555, respectively). These data indicate that phenolic and terpenoid compounds play a major role in antioxidant capacity [36].

Diabetes mellitus is a chronic disease manifested mainly by disorders of carbohydrate metabolism and the number of patients increases each year. Treatment is often expensive and the most appropriate solutions seem to be a well-balanced diet and regular physical effort. Human pancreatic α-amylase and intestinal α-glucosidase are responsible for hydrolyzing carbohydrates into absorbable monosaccharides. The analyzed new artichoke cultivars and hybrids showed significant differences (*p* < 0.05) in their inhibitory activity towards α-amylase and α-glucosidase (Table 4). IC_50_ (mg of dried bracts/mL) for α-amylase and α-glucosidase ranged from 1.38 (“Sambo”) to 3.49 (“Symphony”), and from 1.21 (“Symphony”) to 4.05 (“Calico”), respectively. By definition, IC_50_ is the concentration of substance at which 50% of a specific biological or biochemical function is inhibited. Therefore, the lower is the IC_50_, the lower is the amount of active substance needed to achieve the desired effect. 

Pathogenesis of Alzheimer’s disease correlates with deficiency of acetylcholinesterase (AChE) in the brain. Several clinical trials confirmed that AChE inhibitors may be used to treat this pathology. AChE catalyzes the hydrolysis of acetylocholine (Ach) to choline in order to terminate nerve impulses. Besides AChE, butyrylcholinesterase (BuChE) plays an important role in ACh hydrolysis, especially when a selective anticholinesterase inhibits AChE [37]. IC_50_ inhibition of AChE ranged from 0.09 to 0.12 mg of dried bracts/mL with no significant differences between plant samples (*p* < 0.05).

We also found no significant differences in the values of IC_50_ for BuChE (0.01 to 0.05 mg of dried bracts/mL). Such a low IC_50_ may result from the content of biologically active compounds (Pearson correlation between AChE or BuChE and carotenoids = 0.904 and 0.933, respectively) exhibiting high antioxidant potential (Pearson correlation between AChE or BuChE and ORAC = 0.966 and 0.947, respectively). Our results demonstrated high potential of any cultivar of artichoke as a diet supplement supporting therapy of patients suffering from carbohydrate metabolism disorders and aging adults with neurodegenerative disorders. Multiple studies have proved strong antioxidant and anti-inflammatory activities of polyphenolic compounds (especially flavonoids) that slow down neurodegenerative processes. In addition, in vivo studies on carotenoid and retinoid supplementation revealed their beneficial effect in Alzheimer’s disease [37,38]. Our study is the first to have demonstrated antidiabetic and anticholinesterase activity of selected new artichoke cultivars and hybrids.

### 2.5. Principal Composition Analysis (PCA)

PCA revealed the relationships between antioxidant, antidiabetic and anticholinesterase activity and chemical composition (polyphenolic and terpenoid (carotenoids and chlorophylls) compounds, sugars and organic acids) of new cultivars and hybrids of artichoke cultivated in Spain as was presented on Fig. 3. The first two principal components (PC) explained 62.55% (PC1 = 35.51% and PC2 = 27.04%, respectively) of the total variation of the experimental data. The other principal components with a minor effect on the model were discarded. PC1 was mainly responsible for the differences between the content of phenolic acids, flavonols, carotenoids, chlorophylls and biological activities (including antioxidant capacity, anticholinesterase and α-glucosidase activity). PC2 combined α-amylase activity with the content of organic acids. The analysis revealed differences between artichoke cultivars and hybrids. The highest content of carotenes and chlorophylls in cv. “Blanca de Tudela” mentioned before showed a strong positive correlation with antioxidant activity (ABTS and ORAC). Moreover, significant amounts of phenolic compounds and sugars in “Symphony” strongly correlated with FRAP and α-glucosidase activity. Contrarily, “Sambo” and “Calico”, with lower content of biologically active compounds but high concentration of organic acids demonstrated a stronger correlation with α-amylase activity.

## 3. Materials and Methods

### 3.1. Plant Material and Sample Preparation

Inflorescences of two cultivars (“Blanca de Tudela” and “Calico”) and three hybrids (“Symphony”, “Opera”, and “Sambo”) of artichoke were obtained from an experimental field station of the Miguel Hernández University of Elche (Orihuela, Alicante, Spain).

Bracts were separated from undeveloped artichoke inflorescences. They were ground in a hand mixer (Zelmer; Rzeszów, Poland) and analyzed for dry matter and basic chemical properties (soluble solid, sugar and organic acid content). The bracts frozen in liquid nitrogen and freeze-dried were used for identification and quantification of polyphenolic compounds, carotenoids and for the analysis of the antioxidant potential. The drying process was carried out in a lyophilizer (Alpha 1–4 LSC from Martin Christ GmbH; Osterode, Germany) for 24 h in the following conditions: pressure 0.945 mbar, initial temperature −30 °C, final temperature +30 °C. After drying, the samples were ground in a laboratory mill (type 11A basic from IKA; Staufen, Germany) to a fine powder and stored vacuum-packed in a freezer at −80 °C until analysis.

### 3.2. Basic Chemical Analysis

The dry matter was measured using a vacuum dryer (SPT-200 ZEAMiL Horyzont; Kraków, Poland) according to Wojdyło et al. [39]. The soluble solids content (SSC) was determined in fresh juices from bracts with are refractometer (Atago Rx 5000, Atago Co. Ltd., Kyoto, Japan) and expressed as °Brix. Sugars and organic acids were determined by HPLC-ELSD as described previously by Wojdyło et al. [40], and expressed as g of total sugar content or organic acid per 100 g fresh (FW) or dry weight. All data were obtained in triplicate.

### 3.3. Identification and Quantification of Carotenoids and Polyphenolic Compounds

The extraction and determination of carotenoids and polyphenolic compounds were performed as described previously by Wojdyło, Nowicka and Bąbelewski [20]. Calibration curves for carotenoids and chlorophylls analysis were made from all-trans-β-carotene, chlorophyll a, all-trans-lutein and pheophorbide a. Chlorophyllide and pheophytin derivatives were expressed as chlorophyll a. All incubations were done in triplicate. The results were expressed as milligrams per kilogram of DM. Quantification of polyphenolic compounds was achieved by injection of solutions of known concentrations ranging from 0.05 to 0.5 mg/mL (R^2^ ≤ 0.9998) of chlorogenic acid and luteolin-7-*O*-glucuronide as standards. All data were obtained in triplicate. The results were expressed as mg per 100 g of DM.

### 3.4. Analysis of Biological Activity

Ground dry plant materials (0.5 g) were weighed into a test tube and mixed with 7 mL of 80% aqueous methanol (acidifies with 1% HCl) and the suspension was stirred slightly. Tubes were sonicated twice in Sonic 6D water bath (Polsonic; Warsaw, Poland) for 15 min and extract was centrifuged (5 min, 1000 g).

The ferric reducing ability of plasma (FRAP) and 2,2′-azino-bis(3-ethylbenzothiazoline-6-sulphonic acid) (ABTS) antioxidant assays were determined following Benzie and Strain [41] and Re et al. [42], respectively, using a Shimadzu UV-2401 PC spectrophotometer (Kyoto, Japan). The Oxygen Radical Absorbance Capacity (ORAC) assay was determined on Shimadzu RF-5301 PC spectrofluorometer (Kyoto, Japan) following the method previously described by Ou et al. [43]. All determinations were performed in triplicate. The results were expressed in mM Trolox per 100 g of DM.

The α-amylase and α-glucosidase inhibitory effects of the artichoke leaf extracts were assayed according to the procedure described previously by Wojdyło et al. [20] with slight modifications. The inhibition of AChE and BuChE activity was determined based on Ellman’s method, as reported previously by Wojdyło et al. [40]. All samples were assayed in triplicate and the result was expressed as IC_50_. Analysis was performed using the UV-2401 PC spectrophotometer (Shimadzu; Kyoto, Japan).

### 3.5. Statistical Analysis

Results are given as the mean of three independent determinations (n = 3) ± standard deviation. All statistical analyses and Principal Component Analysis (PCA) were performed with Statistica version 13.3 (StatSoft^®^; Krakow, Poland).

## 4. Conclusions

The research confirmed differences in chemical composition and biological properties of selected cultivars and hybrids of artichoke cultivated in Spain. Cv. “Blanca de Tudela” accumulated the highest content of bioactive compounds and showed the highest antioxidant capacity. The high content of β-carotene and lutein was reflected in the inhibition of AChE and BuChE activity, while the content of polyphenolic compounds had the greatest influence on the antidiabetic activity of the tested cultivars and hybrids. Results of the in vitro trial confirmed that the selected new cultivars and hybrids of artichoke exhibit antidiabetic and anticholinesterase activity. This is an indication to continue this research in vivo.

Therefore, considering the health-promoting effects of phenolic acids, flavonoids and carotenoids, cv. “Blanca de Tudela” has great potential and might be used in the pharmaceutical and food industries to develop functional food products and cosmetics.

## Figures and Tables

**Table 1:** Basic chemical composition of artichoke bracts.

	Blanca de Tudela	Symphony	Opera	Calico	Sambo
**Dry matter (%)**	15.79 ± 0.24 ^c^	14.98 ± 0.09 ^b^	15.37 ± 0.62 ^bc^	13.90 ± 0.02 ^a^	13.74 ± 0.06 ^a^
**SSC (°Bx)**	10.30 ± 0.00 ^a^	10.55 ± 0.07 ^b^	10.70 ± 0.00 ^c^	11.50 ± 0.00 ^e^	11.25 ± 0.07 ^d^
**Sugars (g/100 g FW)**					
Glucose	0.17 ± 0.20	0.47 ± 0.10	0.42 ± 0.22	0.11 ± 0.28	0.20 ± 0.27
Sucrose	0.10 ± 0.44	0.17 ± 0.11	0.21 ± 0.22	0.18 ± 0.18	0.16 ± 0.10
Fructose	0.04 ± 0.91	0.05 ± 0.52	0.03 ± 0.50	0.03 ± 0.55	0.02 ± 0.11
**Total**	0.31 ^a^	0.69 ^b^	0.67 ^b^	0.32 ^a^	0.38 ^a^
**Organic acids (g/100 g DW)**					
Oxalic	0.07 ± 0.01	0.07 ± 0.11	0.06 ± 0.00	0.04 ± 0.00	0.07 ± 0.09
Maleic	0.01 ± 0.00	0.01 ± 0.00	0.01 ± 0.24	0.01 ± 0.00	0.01 ± 0.00
Citric	0.12 ± 0.01	0.02 ± 0.06	0.07 ± 0.09	0.16 ± 0.31	0.10 ± 0.00
Malic	0.22 ± 0.08	0.07 ± 0.00	0.19 ± 0.33	0.44 ± 0.52	0.37 ± 0.28
Quinic	0.09 ± 0.00	0.14 ± 0.01	0.19 ± 0.01	0.07 ± 0.19	0.07 ± 0.37
Shikimic	0.01 ± 0.02	0.02 ± 0.11	0.01 ± 0.11	0.02 ± 0.00	0.02 ± 0.00
Succimic	0.02 ± 0.03	0.06 ± 0.50	0.02 ± 0.25	0.03 ± 0.65	0.02 ± 0.14
Formic	0.09 ± 0.00	0.06 ± 0.41	0.05 ± 0.18	0.09 ± 0.28	0.07 ± 0.17
Adipic	1.21 ± 0.08	1.10 ± 0.12	1.21 ± 0.09	2.20 ± 0.07	1.18 ± 0.00
**Total**	1.84 ^b^	1.50 ^a^	1.81 ^b^	3.05 ^c^	1.18 ^b^

Mean ± standard deviation; in each column different letters denote significant differences between samples (*p* < 0.05).

**Table 2:** Carotenoid and chlorophyll composition in artichoke bracts (mg/kg of dry matter).

Peak No.	R_t_ (min)	Λ_max_ (nm)	[M + H]^+^ (*m*/*z*)	MS/MS (*m*/*z*)	Identified Compounds	Blanca de Tudela	Symphony	Opera	Calico	Sambo
	**Carotenoids**									
1	2.896	412/436/464	601.54	583.53	Neoxantin	8.94 ± 0.00	6.69 ± 0.01	7.19 ± 0.00	2.65 ± 0.02	6.38 ± 0.11
2	5.113	445/474	551.15	476.45	Lutein	131.58 ± 0.00	146.60 ± 0.11	123.42 ± 0.00	62.99 ± 0.00	95.75 ± 0.05
7	8.737	452/478	537.54	444.46	β-caroten	369.61 ± 0.01	274.36 ± 0.00	245.52 ± 0.00	143.04 ± 0.01	208.38 ± 0.01
	**Sum**					510.13 ^e^	427.65 ^d^	376.13 ^c^	208.68 ^a^	310.51 ^b^
	**Chlorophylls**									
3	7.44	453/593/642	907.67	780.69/629.10[M-278]^+^	Chlorophyll b	894.87 ± 0.01	595.38 ± 0.10	697.05 ± 0.22	251.06 ± 0.24	778.08 ± 0.32
4	7.66	433/599/654	885.56	553[M-354 + Na]	Pheophytin b	48.74 ± 0.00	32.01 ± 0.11	35.17 ± 0.02	13.59 ± 0.20	32.04 ± 0.11
5	8.08	430/615/661	892.69	615.13[M-278]^+^/555.34 [M-338]^+^	Chlorophyll a	1250.40 ± 0.05	922.15 ± 0.30	850.07 ± 0.00	380.98 ± 0.65	946.56 ± 0.011
6	8.26	430/615/662	893.69	615.13[M-278]^+^/555.34 [M-338]^+^	Chlorophyll a’	46.25 ± 0.17	37.66 ± 0.03	28.88 ± 0.00	9.57 ± 0.18	21.09 ± 0.09
8	9.14	453/593/642	907.69	629.10 [M-278]^+^	Chlorophyll b’	63.96 ± 0.11	13.42 ± 0.00	88.44 ± 0.35	18.36 ± 0.12	17.90 ± 0.13
9	9.50	433/599/654	885.72	553[M-354 + Na]	Pheophytin b’	24.41 ± 0.09	4.04 ± 0.22	36.94 ± 0.21	3.84 ± 0.00	9.11 ± 0.22
10	9.81	408/503/667	871.72	593.20 [M-278]^+^	Pheophytin a	832.74 ± 0.26	467.94 ± 0.11	776.47 ± 0.19	250.46 ± 0.11	575.96 ± 0.07
11	10.09	408/503/667	871.72	623.36/593.20 [M-278]^+^	Pheophytin a’	90.41 ± 0.07	32.66 ± 0.08	63.20 ± 0.08	20.86 ± 0.26	45.33 ± 0.00
	**Sum**					3251.78 ^e^	2105.26 ^b^	2576.22 ^d^	948.72 ^a^	2426.07 ^c^
	**Total**					3761.91 ^e^	2532.91 ^b^	2952.35 ^d^	1157.40 ^a^	2736.58 ^c^

Mean ± standard deviation; in each column different letters denote significant differences between samples (*p* < 0.05).

**Table 3:** Polyphenolic compounds in artichoke bracts (mg/100 g of dry matter).

Peak No.	R_t_ (min)	Λ_max_ (nm)	[M − H]^−^	MS/MS	Identified Compounds	Blanca de Tudela	Symphony	Opera	Calico	Sambo
1	2.84	326	353.01	190.99	1-*O*-Caffeoylquinic acid	nd	nd	nd	107.32 ± 0.54	230.08 ± 0.00
2	3.31	323	353.01	190.99	3-*O*-Caffeoylquinic acid (neochlorogenic acid)	nd	nd	nd	63.15 ± 0.11	36.04 ± 0.08
3	3.79	330	513.05	338.96/190.99	3-*O*-*p*-Coumaroyl-4-*O*-feruloylquinic acid	nd	nd	nd	0.80 ± 0.88	2.76 ± 0.02
4	4.22	323	353.01	190.99	5-*O*-Caffeoylquinic acid (chlorogenic acid)	62.16 ± 0.22	121.72 ± 0.54	174.45 ± 0.31	1149.06 ± 0.32	390.44 ± 0.00
5	4.50	326	353.01	190.99	4-*O*-Caffeoylquinic acid (cryptochlorogenic acid)	58.67 ± 0.09	62.28 ± 0.22	73.45 ± 0.00	nd	nd
6	4.62	323	705.03	513.00/339.07/191.00	Caffeoylquinic acid dimer	2.42 ± 0.00	1.10 ± 0.75	2.19 ± 0.09	6.71 ± 0.00	nd
7	4.90	367	367.00	191.00	3-*O*-Feruloylquinic acid I	nd	3.81 ± 1,65	3.92 ± 0.05	nd	7.96 ± 0.12
8	5.17	312	337.01	190.99	*p-coumaroylquinic acid*	3.65 ± 0.13	1590.84 ± 0.32	1407.32 ± 0.00	nd	nd
9	5.48	325	513.01	338.96/190.00	4-*O*-*p*-Coumaroyl-5-*O*-feruloylquinic acid	1.44 ± 0.11	0.51 ± 0.12	1.97 ± 0.00	20.92 ± 0.24	8.61 ± 0.09
10	5.53		513.01	338.96/190.00	*p*-Coumaroyl-*O*-feruloylquinic acid I	1826.95 ± 0.01	25.28 ± 0.55	6.34 ± 0.45	nd	6.92 ± 0.07
11	5.60	326	513.01	338.96/190.00	*p*-Coumaroyl-*O*-feruloylquinic acid II	16.98 ± 0.28	1.10 ± 0.00	1.05 ± 0.37	nd	nd
12	5.81	326	515.01	190.99	di-Caffeoylquinic acid I	1.35 ± 0.66	19.91 ± 0.11	0.82 ± 0.13	nd	nd
13	6.30	326	367.00	191.00/178.97	3-*O*-Feruloylquinic acid II	15.44 ± 0.21	12.42 ± 0.05	17.82 ± 0.11	nd	4.19 ± 1.12
14	6.90	325	515.01	323.00/190.99	di-Caffeoylquinic VI	3.89 ± 1.14	2.18 ± 0.27	4.09 ± 1.21	nd	1.41 ± 0.32
16	7.43	326	515.01	353.01/190.99/179.00	di-Caffeoylquinic acid II	0.72 ± 0.65	9.63 ± 0.14	5.50 ± 0.18	nd	692.56 ± 0.45
18	7.67	327	515.01	353.00/190.99	di-Caffeoylquinic acid III	nd	2.19 ± 0.02	nd	12.10 ± 0.08	69.00 ± 0.15
19	7.71	327	515.01	353.00/190.99	di-Caffeoylquinic acid IV	2.17 ± 0.25	6.73 ± 0.02	3.55 ± 0.03	1244.44 ± 0.07	110.11 ± 0.16
22	8.25	325	515.01	190.99/179.00/172.98	di-Caffeoylquinic acid V	11.59 ± 0.09	1577.93 ± 0.00	1561.85 ± 0.65	8.36 ± 0.00	6.53 ± 0.02
	**Sum**					2007.42 ^b^	3437.64 ^d^	3264.32 ^d^	2618.88 ^c^	1567.60 ^a^
15	7.20	254, 268	461.09	285.09	Luteolin-7-*O*-glucuronide	5.58 ± 0.65	4.18 ± 1.22	8.37 ± 0.46	nd	40.93 ± 0.11
17	7.58	345	431.09	269.00	Apigenin-7-glucoside	3.33 ± 0.50	10.85 ± 0.69	12.27 ± 0.27	75.51 ± 0.01	15.77 ± 0.11
20	7.98	271, 341	593.08	285.09	Luteolin-7-rutinoside (scolymoside)	71.34 ± 0.11	77.08 ± 0.36	95.11 ± 0.06	7.19 ± 0.25	44.00 ± 0.32
21	8.10	265, 339	445.09	269.09	Apigenin-7-glucuronide	36.48 ± 0.09	106.45 ± 0.12	44.61 ± 0.00	12.74 ± 0.03	13.19 ± 0.54
23	8.44	265, 339	596.01	269.07	Derivatives of apigenin	3.85 ± 0.22	nd	9.39 ± 0.19	nd	nd
24	8.49	265, 339	543.02	269.07	Derivatives of apigenin	8.42 ± 0.36	2.71 ± 0.22	nd	42.51 ± 0.41	nd
	**Sum**					129.01 ^b^	201.27 ^d^	169.75 ^c^	137.95 ^b^	113.89 ^a^
	**Total**					2136.43 ^b^	3638.91 ^e^	3434.07 ^d^	2756.83 ^c^	1681.49 ^a^

Mean ± standard deviation; nd, not detected; in each column different letters denote significant differences between samples (*p* < 0.05).

**Table 4:** Antioxidant (mmol Trolox/100 g), antidiabetic and anticholinoesterase (IC_50_; mg/mL) activity of artichoke bracts.

Cultivar/Hybrid	Antioxidant Activity			Antidiabetic Activity		Anticholinoesterase Activity	
	ABTS	FRAP	ORAC	α-amylase	α-glucosidase	AChE	BuChE
**Blanca de Tudela**	20.74 ± 0.75 ^c^	9.15 ± 0.34 ^a^	39.98 ± 1.21 ^e^	3.20 ± 0.02 ^c^	2.51 ± 0.29 ^b^	0.09 ± 0.05 ^a^	0.07 ± 0.04 ^a^
**Symphony**	15.80 ± 1.15 ^b^	13.78 ± 0.85 ^d^	34.16 ± 3.14 ^d^	3.49 ± 0.28 ^d^	1.21 ± 0.04 ^a^	0.09 ± 0.05 ^a^	0.08 ± 0.04 ^a^
**Opera**	16.73 ± 0.45 ^b^	11.94 ± 0.68 ^c^	26.77 ± 0.53 ^c^	2.74 ± 0.43 ^c^	3.31 ± 0.26 ^c^	0.11 ± 0.06 ^a^	0.08 ± 0.05 ^a^
**Calico**	12.49 ± 0.51 ^a^	10.33 ± 0.56 ^b^	21.45 ± 1.35 ^b^	2.22 ± 0.39 ^b^	4.05 ± 0.05 ^e^	0.12 ± 0.06 ^a^	0.09 ± 0.05 ^a^
**Sambo**	17.20 ± 1.02 ^b^	11.35 ± 0.67 ^bc^	16.80 ± 1.35 ^a^	1.38 ± 0.28 ^a^	3.55 ± 0.20 ^d^	0.12 ± 0.01 ^a^	0.09 ± 0.01 ^a^

Mean ± standard deviation; in each column different letters denote significant differences between samples (*p* < 0.05).
